# Designing Area Optimized Application-Specific Network-On-Chip Architectures while Providing Hard QoS Guarantees

**DOI:** 10.1371/journal.pone.0125230

**Published:** 2015-04-21

**Authors:** Sajid Gul Khawaja, Mian Hamza Mushtaq, Shoab A. Khan, M. Usman Akram, Habib ullah Jamal

**Affiliations:** 1 Department of Computer Engineering, National University of Sciences and Technology, Islamabad, Pakistan; 2 Ghulam Ishaq Khan Institute, Topi, Pakistan; Southwest University, CHINA

## Abstract

With the increase of transistors' density, popularity of System on Chip (SoC) has increased exponentially. As a communication module for SoC, Network on Chip (NoC) framework has been adapted as its backbone. In this paper, we propose a methodology for designing area-optimized application specific NoC while providing hard Quality of Service (QoS) guarantees for real time flows. The novelty of the proposed system lies in derivation of a Mixed Integer Linear Programming model which is then used to generate a resource optimal Network on Chip (NoC) topology and architecture while considering traffic and QoS requirements. We also present the micro-architectural design features used for enabling traffic and latency guarantees and discuss how the solution adapts for dynamic variations in the application traffic. The paper highlights the effectiveness of proposed method by generating resource efficient NoC solutions for both industrial and benchmark applications. The area-optimized results are generated in few seconds by proposed technique, without resorting to heuristics, even for an application with 48 traffic flows.

## 1 Introduction

As CMOS technologies have scaled down, increasingly large system-on-chip (SoC) designs are being manufactured today. With high demands for system performance, Multiprocessor System on Chip (MPSoC) architectures with a large count and variety of modules such as processors, external I/O interfaces and application cores are being developed. Interconnect architectures are required for these systems which can scale to meet their inter-module communication requirements while keeping system costs in check. Network-on-chips (NoC) have been presented as a structured solution to meet these needs and have been a focus of active research and development in recent years as a scalable alternative to buses and crossbars.

The communication characteristics of a SoC are either determinable at design time or dynamically generated during run time. For application-specific SoCs, the traffic requirements for the application can be statically determined based on task mappings, data flow graphs or application communication traces. In addition to the bandwidth specifications for the communication, there may be real-time constraints for the application with hard or soft latency bounds on the traffic flows.

While design automation tools have been developed for application specific NoC design in both industry and academia, they have generally focused in one of two directions. One area of focus has been on the generation of application specific NoC topologies which meet or optimize cost requirements and cater to the application bandwidth specifications. However, latency deadline requirements in the design flow are either ignored or handled in a post-topology generation step by iteratively tuning the resources until all requirements are met, leading to a less than optimal architectural solution. To scale for large designs, these design flows may also resort to heuristics for generation of a good enough application specific NoC topology.

The other direction where research has concentrated is based on utilizing either a manual topology specification or a regular topology (eg. Mesh, Torus) for the NoC architecture and then use some form of resource reservation to meet the traffic and latency requirements of the application. These topologies may not necessarily be optimized for cost and the manual specification of the topology may also require extra effort. Further, the resource reservation techniques utilized to cater for worst-case deadline scenarios of the application are mostly based on inflexible forms of resource reservation. So they are not adaptive to flexible and slight changes in traffic patterns.

In this work we propose a methodology for designing optimized application specific network-on-chip topologies and generating efficient architectures while meeting hard QoS requirements of the application simultaneously. More specifically, the proposed system presents a mathematical model which generates an area-optimized network-on-chip topology given a set of bandwidth and QoS of the application. The paper extends the model so that it can work with two types of NoC architectures. We also present micro-architectural techniques on how proposed system incorporates QoS in the network-on-chip while efficiently utilizing hardware resources and keeping the solution flexible for dynamic traffic variations. It should be noted that power minimization is only indirectly considered in the solution as explained in section 3.

The remainder of the paper is organized as follows. Section 2 reviews the existing related work to our methodology. Section 3 discusses the architectural aspects of the design methodology. The formal introduction of problem and presentation of Mixed Integer Linear Programming (MILP) model is presented in section 4. Section 5 shows the results of our technique with two design examples followed by conclusion in last section.

## 2 Related Work

Different micro-architectural mechanisms for enabling guaranteed traffic in the NoC routers have been presented in literature. Time division multiple access (TDMA) approaches work by using schedules to avoid contention within the network, but do not adapt to changes in traffic such as jitter [1]. Virtual channels, circuit switching and prioritization mechanisms are also options used to provide guarantees for traffic flows [2]. However, these works do not show how NoC solution overall can be resourced efficiently for application specific demands. A state-of-the-art end to end design flow using manual or regular topology and guaranteeing resources can be found in [3].

A number of techniques on generating application specific network-on-chip topologies have been proposed [4–12]. Sirnivasan et al. [4] presented a MILP formulation to generate application specific topology while attempting to minimize power indirectly. However, they restricted the dimension and structure of routers for any topology to a single type such as every router in the network should have only three input and output ports. This approach limited the possible solutions which can be assessed and thus may not generate the best possible topology for the application. While the method did not provide latency bounds, latency constraints are shown to be handled based on the number of hop-counts between source and destination by limiting number of input links for an output edge to restrict packet contention which added further constraints to the topology solution. For large designs, they proposed the use of heuristic solutions instead of the MILP formulation.

A method for MILP formulation is proposed in [5] which attempted to optimize area and frequency efficiency and supported flexible number of ports for individual routers. The work is focused on cascaded-cross bars and did not cover the problem of contention between competing packets for the same link. Thus no bounds on the latency for guaranteed traffic can be provided through this method. The MILP solution also takes long time to solve for the given examples. The method in [7] also provided a MILP formulation for power optimized NoC topology generation by keeping contention or QoS into consideration.

Atienza et al. [11] presented an algorithm which creates a low power-application specific NoC. Latency is handled by a mismatch parameter which is adjusted in the formulation through an iterative procedure by observing simulations of a previous generated topology. This may be a lengthy procedure and the mismatch parameter may lead to additional unnecessary resources. Further, the acceptance of delays is dependant on the simulated test cases alone, which may or may not cover all contention based corner cases.

Another method which considers the problem of contention while generating application specific NoC topologies using a tabu search method to minimize power is proposed in [12]. As part of the topology generation step, they ask the user to input the arbitration delay at every node. This however does not account for delays due to blocking probabilities along the path. The work uses a layered contention model to analyze points of contention which cause the blocking in order to relieve them by insertion of virtual channels, trading power and area costs for performance. Delay calculations through the model however are based on average estimates of traffic arrival times and delays to estimate power and do not provide strict latency bounds for worse case latency scenarios.

In terms of calculating latency guarantees for a given fixed topology, Hansson et al. [13] used a latency-rate server model to calculate the minimum size of the network interface buffers which would enable application specific performance guarantees. However the work doesnt address the limitations of using a latency rate server model for calculating delays which is that the latency bounds are quite loose. This thing has been highlighted in [14] where the authors showed how to find tight bounds by taking into account delays caused by contention in aggregate multiplexing in an optimization problem. The formulation of the mathematical model is such that it isn’t trivial to extend the solution within an optimization formulation which would generate application specific topologies.

The calculations related to worst-case delays for NoCs are presented in [15]. This work has been extended in [16] where it has been embedded as part of a greedy algorithm and an application specific Noc generation technique is presented which provides QoS guarantees. The method is based on calculating the worst possible duration which all flows in a topology can face during arbitration at every node and is calculated with an assumption of round-robin arbitration. This calculation is done for every generated topology and is fed back into the algorithm until a least-cost solution which meets the worst case scenarios for guaranteeing latency bounds. While selection of the round-robin arbiter enables contention delays to be calculated, it adds additional restrictions to the normal flow restrictions which dictate aggregate flow through an output cannot exceed capacity. With a round-robin arbiter, the maximum percentage of time an output link can be utilized by an input port becomes (1/n)*100, where n inputs links compete for the same output link of a router. This puts a limitation on multiplexing many flows together through an output link and especially so when a flow has relatively high throughput traffic. Further, as the method leads to flow path assignment for worst-case contention scenarios it leads to utilizing extra routers, resources, and/or higher frequency and power costs, to cater for the traffic requirements in the resulting network-on-chip. The authors do not provide any power or area costs of the solution generated after applying the contention model.

The novelty of proposed method is that instead of catering for worst case contention, it models the path assignments and uses a static priority mechanism in the router micro-architecture so flows with real-time requirements do not face contention. The proposed system also foregoes static timeslot reservation on the router so that remaining flows are not blocked and the router adapts to traffic conditions for maximum link utilization as discussed in section 3. The combination of our router design and modeling rules enable the generation of a resource-optimized NoC for the given bandwidth and latency requirements.

## 3 Proposed Architecture

We begin with an overview of the key design features we require from an optimized network-on-chip and then describe the design decisions we made to accommodate those features. A more formal problem for the generation of optimized topology description is introduced later in Section 4. The key features are as follows:

1. The solution should provide latency guarantees where required.
2. The throughput requirements have to be met, not only for guaranteed flows, but for all flows as specified by the application traffic requirements.
3. Minimum restrictions should be applicable to the router dimensions as well as the network topology due to the router architecture.
4. Latency guarantees mechanisms have to be resource efficient.
5. The solution should aim to have high link utilization in terms of traffic flow assignments to link.
6. Packets should face minimum slack due to arbitration or scheduling.
7. Resources used for virtual channels and buffers should be kept at a minimum in the routers.

The steps to fulfill these requirements using proposed solution are explained here.

### 3.1 Latency Guarantees

The proposed solution to provide hard latency guarantees for an optimized NoC is to utilize a prioritized traffic mechanism, and this logic is then embedded in the mathematical model while generating path assignments for the optimized NoC topology. Thus, the NoC supports a differentiated service with two classes of traffic, with one class having priority over the other. If an output link at a router has a packet from the priority flow, it will stall any competing packet for the output link to forward the prioritized packet. As it wins any contention with other packets for any output link and has no setup time, the latency through the network for the packets is the same as the hop-count of the path assigned to the flow in the network. Further, no restrictive resource reservation is required for the traffic flow along its path as other flows are free to use the network when there are no priority packets flowing. We note that the underlying assumption which enables us to use this solution is that the number of flows which will require latency guarantees is a smaller number than the best effort flows. Therefore, the proposed system multiplexes both real-time and best effort flows through the same output links with the exception of assigning multiple real-time flows through the same path so that they do not contend with each other.

### 3.2 Throughput regulation

Throughput specifications of traffic flows of the application have to be met by the generated solution. The mathematical model assigns multiple flows through links and provides an aggregate guarantee on the bandwidth availability of any link so that it does not exceed capacity over a period of time. The arbiter design within the router then has to functionally match these requirements as the traffic may be bursty or aperiodically scheduled [17]. We combine two techniques together so the resulting arbiter regulates the throughput to match the requirements for flows with or without hard latency guarantees.

#### 3.2.1 Rate based scheduling

We use a rate based scheduler along with static priority based arbitration logic to regulate traffic. The technique works by having a set number tokens assigned to each flow in the beginning of a period of time in proportion to relative bandwidth share of the contending link within that period. Each packet sent by a particular traffic flow leads to the reduction of a token available to that flow, and when the tokens for a flow finish further packets for that flow cannot traverse further within the set period. This period is also calculated by a number of tokens which equal to the number of cycles which comprises a period and the tokens are reduced by one every cycle and resets when it reaches zero. After the period is over, the number of tokens is also reset for each flow. Arbitration between competing flows with both tokens and flits to be sent forward at a given cycle is done on the basis of static priority between the flows. The rate based scheduling helps to allocate flows to the links based on their relative traffic requirements. At the same time, using static priority does not place strict scheduling requirements for the traffic pattern or advance resource reservation. Finally, keeping a static priority also keeps the realized logic simple and so results in low area usage.

#### 3.2.2 Slack based adaptation

While the rate based mechanism is adaptive based on what packets are available in the input, an outgoing link may still face some under utilization in some cases. For example, if the only packet available at input for a port is from a flow which has already used up its tokens for the period, the packet would be blocked while the output link would be unused. We modify the arbiter design above to reduce this slack by enabling packets to be forwarded for a flow even when all the tokens for that flow in the period have been utilized, provided that there are no immediate competing packets with available tokens or having a higher. This helps to reduce the under utilization of the output links, while the additional logic for this is simple and resource efficient.

### 3.3 Area-efficient micro-architecture

#### 3.3.1 Single-flit buffers

We use worm-hole based flow control in the routers, allotting a single flit buffer to each of the flows through the router. The single flit buffer helps to keep the area cost of the solution low, as well as implicitly reduce power consumption as large buffers and virtual channels are removed.

#### 3.3.2 Single flow links

From the optimized topology result, if any link is utilized by only a single flow, we replace that network link with a point-to-point connection, as lack of flow multiplexing means that an the router is not needed and is only consuming extra power and area.

### 3.4 Cost Modeling

Our works focuses on minimizing the area cost of the application specific network on chip. Specifically, we have modeled and optimized the costs for FPGA based network-on-chips for our examples.

#### 3.4.1 Router area cost model

For evaluating the cost model of the network-on-chip, we use linear regression on various instantiated routers with different dimensions to generate a router area cost based on the number of flows connecting with output links, bit-width etc. As the synthesis tool available to us was for FPGAs, for experimental purposes we model the router area in terms of slice registers and slice LUTs. This is different from the traditional number of utilized slice used to discuss FPGA area utilized, and is done so that the model generates results based on the synthesized logic and not on any single platform implementation. We get linear equations for both slice registers and slice LUTS based on the number of flows every router output link handles, which we combine into a single linear equation which is used in the model which will be discussed in Section 4. For evaluating the costs, we build our own low-area cost network-on-chip generation tool.

#### 3.4.2 Power model

The mathematical models (Section 4) do not explicitly model power cost of the network on chip. The power cost of network-on-chip solution is dependant on the micro-architecture, topology and floor planning. As discussed in [18], high level modeling for power for a particular topology instance requires knowledge of the time a packet is being transmitted or being blocked at a router. This information therefore has to be derived from not just a worst-case contention model but an average delay model at each router along the paths to generate power analysis for the solutions. We do not model this as our work is focused on area-optimized NoC and so it is beyond the scope of our work. However, by generating a resource efficient topology and architecture for the NoC, we are implicitly reducing the power cost and thereby providing for a power-efficient NoC solution.

### 3.5 Network-on-chip Topology Architectures

The solution to the problem of generating optimized topologies may entail different topological and micro architectural constraints based on possible choices available by the designer. We present an overview of two types of network on chip solutions for which we will be subsequently presenting mixed integer linear programming formulations. Our mathematical model is constructed in such a way that it is easy to adapt it for the two types which are discussed next.

#### 3.5.1 Type A: NoC with multi-port network interface

**Fig 1:**
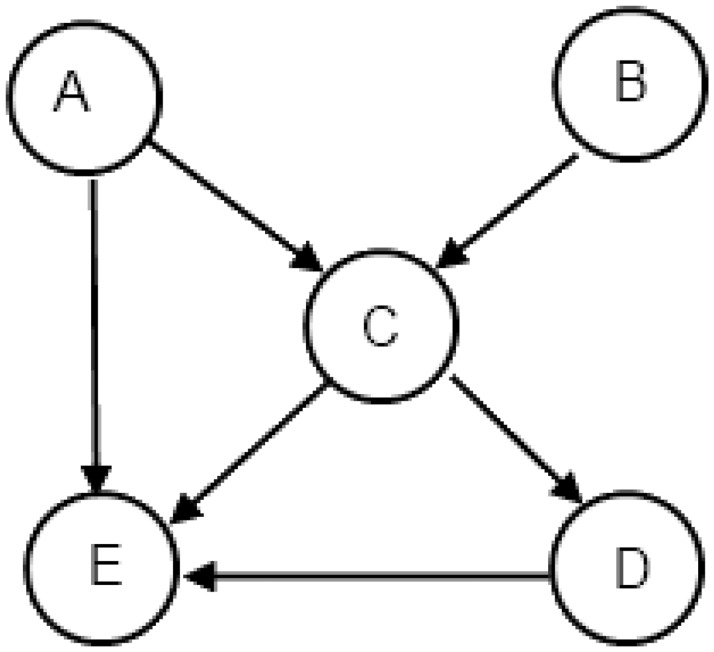
Sample Application Traffic Connectivity.

**Fig 2:**
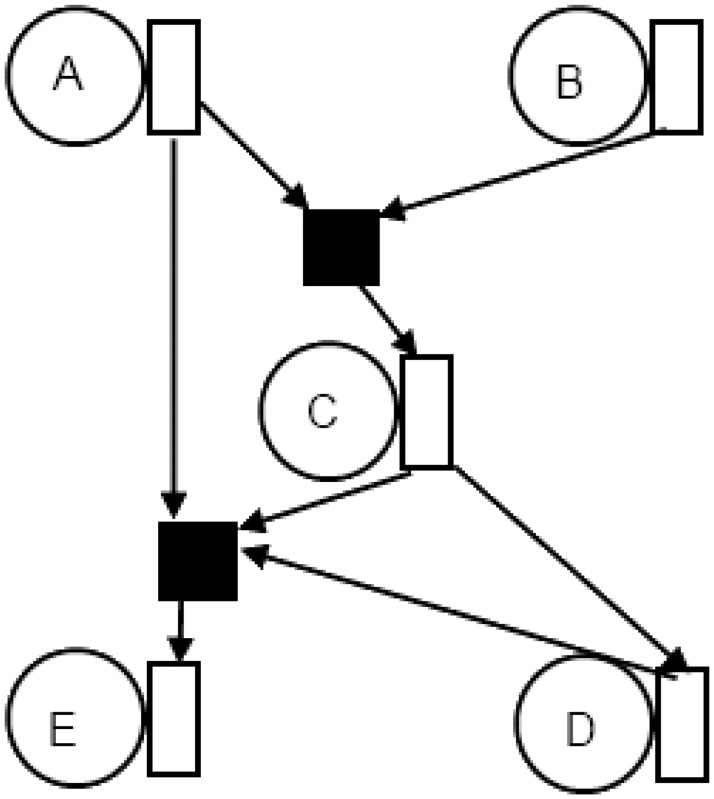
Node to router connectivity for Type A architecture.

Our first architecture considers that every node has a network interface which further connects to multiple routers. By allowing the network interface (NI) to connect to various routers, an area optimized topology solution will be such that only a single router will exist between every source network interface and destination network interface. Essentially, the routers are shared between different nodes. The second feature of this topology is that each router has only a single output port while supporting multiple input ports. Fig 1 shows a traffic connectivity graph of a sample application. Fig 2 shows the realization of traffic requirement of various nodes from Fig 1 as Type A NoC topology architecture. Here nodes can be any processing entity and directed links show the movement of data between these entities. The black colored filled boxes in the figures represent the routers. The unfilled box with each node is the network interface (NI).

#### 3.5.2 Type B: NoC with multi-port local routers

**Fig 3:**
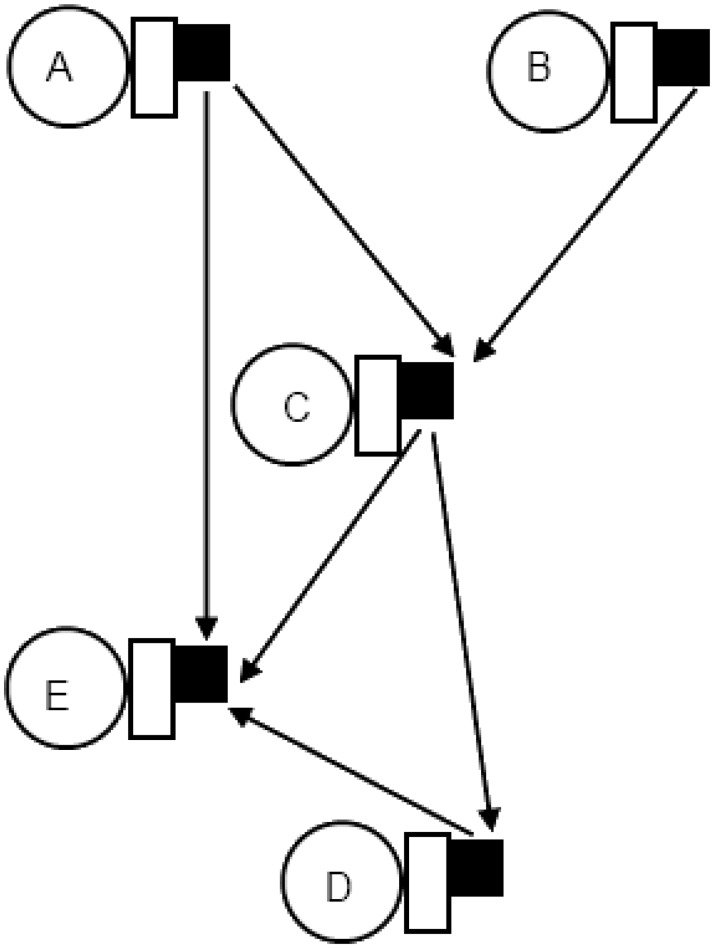
Node to router connectivity for Type B architecture.

The second topology architecture restricts every network interface to a single router, thereby providing every node with a local router. Further, we do not include any routers in the topology which are not connected to a node. An area-optimized architecture results in the local router of the source connecting directly to the local router of the destination node. Fig 3 shows an example of the application given in Fig 1 for type B architecture.

## 4 Mixed integer linear programming model

### 4.1 Problem

We begin with a formal definition of the problem using the concepts used in [4] and [5]. Initially an undirected communication trace graph *G*(*V*_*m*_, *V*_*s*_, *E*_*e*_) of the given application is acquired from a specific application. Here *v*_*m*_ *ϵV* denotes a master node, *v*_*s*_ *ϵV* denotes a slave node, and *e*_*z*_ *ϵE* denotes an undirected edge between *v*_*m*_ and *v*_*s*_ representing flow of traffic from *v*_*m*_ to *v*_*s*_. To formulate the MILP model for specific application, following requirements are gathered

1. For every *e*_*z*_ *ϵE*, *T*(*e*_*z*_) represents the bandwidth requirement of *e*_*z*_ in mb/s.
2. For every *e*_*z*_ *ϵE*, *Q*(*e*_*z*_) is a binary parameter for which the value of 1 represents a priority for low latency of the traffic flow and a value of 0 means that the flow does not require such a priority.
3. For applications with multiple modes, we assume that the communication trace graph has been specified to include the maximum traffic values from all application modes.

We also add the following constants for the MILP model:

1. System frequency (*f*) in megahertz
2. Router data bit-width (*b*)

Other than these requirements, following assumptions are also made

1. Let the maximum number of routers (*rϵR*) which can utilized by the NoC be equal to the number of edges in the communication trace graph. The reason for the limit is that it represents the case where no flow is multiplexed with any other flow through a router, and there is no unused router.
2. Let us also declare three binary decision variables which define the topology of the solution that we require.
  - *RR*_*e*,*r*,*r*′_, *eϵE*, *rϵR*, *r*′ *ϵR* defines the set of router to router paths traversed by the flow e. *r* = *r* represents the case where a single router is traversed by the flow.
  - *MR*_*e*,*r*_, *eϵE*, *rϵR* is used to define the first router from which the flow e traverses into the network from the master node
  - *LR*_*e*,*r*_, *eϵE*, *rϵR* is used to define the last router from which the flow e traverses outside of the network to the slave node.

The problem is to find the topology *T*(*MR*, *LR*, *RR*) which provides the least area cost while subject to the bandwidth requirements and other constraints listed (such as frequency) or later introduced.

### 4.2 MILP formulation for TYPE A

#### 4.2.1 Path constraints

These constraints restrict that for each flow the packets traverse into the network through only router as well as traverse out of the network through only one router.

$$\begin{array}{c} \forall \;e\epsilon E\;\Sigma_{r\epsilon R}\;MR_{e,r}=1 \end{array}$$ (1)

$$\begin{array}{c} \forall \;e\epsilon E\;\Sigma_{r\epsilon R}\;LR_{e,r}=1 \end{array}$$ (2)

The following two constraints establishes that only a single router paths are considered by each flow between the master and slave nodes for the Type A case.

$$\begin{array}{c} \forall \;e,r,e\epsilon E,r\epsilon R,r^{\prime }\epsilon R,r=r^{\prime }\;\Sigma_{r^{\prime }\epsilon R}RR_{e,r,r^{\prime }}RR_{e,r,r^{\prime }}\;<=\;1 \end{array}$$ (3)

$$\begin{array}{c} \forall \;e,r,e\epsilon E,r\epsilon R,r^{\prime }\epsilon R,r!=r^{\prime }\;\Sigma_{r^{\prime }\epsilon R}RR_{e,r,r^{\prime }}RR_{e,r,r^{\prime }}\;<=\;0 \end{array}$$ (4)

The following constraints establish the paths from the master node through the network to the slave node. Eq (5) established the connection through a weight based model.

$$\begin{array}{c} \forall e,r,e\epsilon E,r\epsilon R\;-MR_{e,r}+LR_{e,r}\;=\;0 \end{array}$$ (5)

We also need to for the variable RR to establish a path through a single link router only if both the master and slave nodes are connected to the same router for the given flow ie:

$$\begin{array}{c} \forall e,r,e\epsilon E,r\epsilon R\;RR_{e,r,r^{\prime }}\;=\;MR_{e,r}*LR_{e,r} \end{array}$$ (6)

However, Eq (6) is a non-linear equation. So instead of using Eq (6), we model the constraint in a linear form as follows:

$$\begin{array}{c} \forall e,r,e\epsilon E,r\epsilon R\;MR_{e,r}+LR_{e,r}\;>=\;2*RR_{e,r,r^{\prime }} \end{array}$$ (7)

$$\begin{array}{c} \forall e,r,e\epsilon E,r\epsilon R\;1-MR_{e,r}+LR_{e,r}\;=\;2*RR_{e,r,r^{\prime }} \end{array}$$ (8)

Thus Eqs (7) and (8) then act as the linear equivalent of Eq (6)

#### 4.2.2 Flow constraints

Next we route the given traffic flows through the links so that the sum of average bandwidth for all links does not exceed their capacity. First we define a variable ST which represents the assigned bandwidth for all traffic flows through each link between a maximum value of 1 and a minimum of zero.

$$\begin{array}{c} ST_{r,r^{\prime }},r\epsilon R,r^{\prime }\epsilon R \end{array}$$

$$\begin{array}{c} \forall r,r\epsilon R\;0<=ST_{r,r}\;<=\;1 \end{array}$$ (9)

Next, using the declared parameters for bit-width and frequency, we set the capacity limits for flow assignments to the routers. Our assumption is that the bit-width and frequency are set such that the destination node can receive the average traffic bandwidth as specified in the communication task graph.

$$\begin{array}{c} \forall r,r\epsilon R\;ST_{r,r^{\prime }}\;=\;\Sigma_{e\epsilon E}RR_{e,r,r^{\prime }}\;*\left(T_{e\epsilon E}*b/f\right) \end{array}$$ (10)

#### 4.2.3 QoS constraints

Next we add flow restrictions for priority flows, if and where applicable based on the given traffic requirements, such that one link has only one priority flow routed through it at any time. Based on our assumptions of bit-width and frequency as part of the flow constraints, we check before running the model that the latency constraints will be met as part of a two-hop route.

$$\begin{array}{c} r,r\epsilon R\;\Sigma_{e\epsilon E}RR_{e,r,r^{\prime }}\;*Q_{e\epsilon E}M\;<=\;1 \end{array}$$ (11)

$$\begin{array}{c} r,r\epsilon R\;\Sigma_{e\epsilon E}MR_{e,r}\;*Q_{e\epsilon E}M\;<=\;1 \end{array}$$ (12)

$$\begin{array}{c} r,r\epsilon R\;\Sigma_{e\epsilon E}SR_{e,r}\;*Q_{e\epsilon E}M\;<=\;1 \end{array}$$ (13)

#### 4.2.4 Cost evaluation

We next discuss how to calculate the cost of the network which is a minimization goal for the model. The general router costs are initially generated by linear regression as discussed earlier. For a particular selected bit-width, all flows routed through a particular output link of a router generate a router cost of

$$\begin{array}{c} Cx+D \end{array}$$ (14)

where X is the number of flows, and *C* and *D* are constants. Thus to find the aggregate router costs in the solution, we need to sum together the number of flows assigned to each output link utilized by the solution as well as the total number of output links.

Let’s define the following variables for this purpose: FPO, TF, OU, TO

*FPO*_*r*,*r*′_, *rϵR* is an integer variable which holds the number of flows assigned to each output link as shown in Eq (15)

$$\begin{array}{c} \forall r,r^{\prime }\epsilon R\;FPO_{r,r^{\prime }}\;=\;\Sigma_{e\epsilon E}RR_{e,r,r^{\prime }} \end{array}$$ (15)

*TF* is an integer variable which holds the total sum of for all output links.

$$\begin{array}{c} TF\;=\;\Sigma_{r\epsilon E\;FPO_{r,r^{\prime }}} \end{array}$$ (16)

*OU*_*r*,*r*′_, *rϵR* is a binary variable which is 1 if an output link is assigned any flow in the solution and 0 if it is not. This is set by the two equations below.

$$\begin{array}{c} \forall r,r^{\prime }\epsilon RFPO_{r,r^{\prime }}*0.0001\;<=\;OU_{r,r^{\prime }} \end{array}$$ (17)

$$\begin{array}{c} \forall r,r^{\prime }\epsilon RFPO_{r,r^{\prime }}*0.0001\;<=\;OU_{r,r^{\prime }} \end{array}$$ (18)

Note that the constant in Eq (17) has to be sufficiently low so that multiplication with the variable results in a value below 1 unless the variable is 0. Similarly the constant in Eq (18) has to be such that multiplication with the variable results in a value larger than 1 unless the variable is zero.

*TO* is an integer variable which holds the total sum of *OU* for all output links.

$$\begin{array}{c} TF\;=\;\Sigma_{r\epsilon E}OU_{r,r^{\prime }} \end{array}$$ (19)

Thus we derive from Eq 14 the total router cost, which is the objective to be minimized, follows:

$$\begin{array}{c} TO*C\;+\;TF*D \end{array}$$ (20)

### 4.3 Milp Formulation for Type 2 Architecture

Unless this section mentions equations as dropped, modified or updated, all equations from the Type 1 formulation are applicable for Type 2 formulation. For the Type 2 formulation, we first need to assign a local router for each of the nodes. For every flow of a node, we assign constraints on *LR* if the node is receiving a flow and on *MR* if it is the source of the flow.

$$\begin{array}{c} LR_{e_{i},r_{j}}\;=\;1 \end{array}$$ (21)

$$\begin{array}{c} MR_{e_{i},r_{k}}\;=\;1 \end{array}$$ (22)

We also remove Eq (12) from the formulation as one master node may be the source of multiple priority flows and consequently so may be the local router connected to the source node. However, we retain Eq (13) as per our definition of the priority, only one flow will have priority at a destination node, for which it faces no contention.

Next we modify Eqs (3) and (4) so that the case of only one router in the network is not considered, and instead multi-router links are considered. This is done as follows:

$$\begin{array}{c} \forall e,r,e\epsilon E,r\epsilon R,r^{\prime }\epsilon R,r=r^{\prime }\;\Sigma_{r^{\prime }\epsilon R}RR_{e,r,r^{\prime }}=0 \end{array}$$ (23)

$$\begin{array}{c} \forall e,r,e\epsilon E,r\epsilon R,r^{\prime }\epsilon R,r!=r^{\prime }\;\Sigma_{r^{\prime }\epsilon R}RR_{e,r,r^{\prime }}<=1 \end{array}$$ (24)

Eq (5) needs to be update to included router to router links. For this we define two binary variables *FR*_*e*,*r*_ and. For every flow and router, has a value of 1 if a flow goes from the router to another router. Similarly for every flow and router *BR*_*e*,*r*_ has a value of 1 if a flow comes from another router to the router and 0 if it does not. This is set by the following two equations:

$$\begin{array}{c} \forall e,r,e\epsilon E,r\epsilon R,r^{\prime }\epsilon R,r!=r^{\prime }\;FR_{e,r}\;=\;\Sigma_{r^{\prime }\epsilon R}RR_{e,r,r^{\prime }} \end{array}$$ (25)

$$\begin{array}{c} \forall e,r,e\epsilon E,r\epsilon R,r^{\prime }\epsilon R,r!=r^{\prime }\;BR_{e,r}\;=\;\Sigma_{r\epsilon R}RR_{e,r,r^{\prime }} \end{array}$$ (26)

Then Eq (5) is updated with the new variables to create the following flow based equation for the router connectivity for each flow. This also helps keep the a single path from source to destination for each flow

$$\begin{array}{c} \forall e,r,e\epsilon E,r\epsilon R\;-MR_{e,r}+FR_{e,r}-BR_{e,r}+LR_{e,r}\;=\;0 \end{array}$$ (27)

Lastly, the Eqs (9, 10, 11, 15, 16, 17, 18 and 19) are updated to consider multiple router links and not consider single router links, leading to the following b versions of the equations.

$$\begin{array}{c} \forall r,r^{\prime },r\epsilon R,r^{\prime }\epsilon R,r!=r^{\prime }\;\;0<=\;ST_{r,r^{\prime }}<=1 \end{array}$$ (9b)

$$\begin{array}{c} \forall r,r^{\prime },r\epsilon R,r^{\prime }\epsilon R,r!=r^{\prime }\;\;ST_{r,r^{\prime }}=\Sigma_{e\epsilon E}RR_{e,r,r^{\prime }}*\left(T_{e\epsilon E}*b/f\right) \end{array}$$ (10b)

$$\begin{array}{c} \forall r,r^{\prime },r\epsilon R,r^{\prime }\epsilon R,r!=r^{\prime }\;\Sigma_{e\epsilon E}RR_{e,r,r^{\prime }}*Q_{e\epsilon E}M\;<=\;1 \end{array}$$ (11b)

$$\begin{array}{c} \forall r,r^{\prime },r\epsilon R,r^{\prime }\epsilon R,r!=r^{\prime }\;FPO_{r,r^{\prime }}\;=\;\Sigma_{e\epsilon E}RR_{e,e,r^{\prime }} \end{array}$$ (15b)

$$\begin{array}{c} TF\;=\;\Sigma_{r\epsilon E}FPO_{r,r^{\prime }}S \end{array}$$ (16b)

$$\begin{array}{c} \forall r,r^{\prime },r\epsilon R,r^{\prime }\epsilon R,r!=r^{\prime }\;FPO_{r,r^{\prime }}*0.0001<=OU_{r,r^{\prime }} \end{array}$$ (17b)

$$\begin{array}{c} \forall r,r^{\prime },r\epsilon R,r^{\prime }\epsilon R,r!=r^{\prime }\;FPO_{r,r^{\prime }}*1.1>=OU_{r,r^{\prime }} \end{array}$$ (18b)

$$\begin{array}{c} TF\;=\;\Sigma_{r\epsilon E,r^{\prime }\epsilon E}OU_{r,r^{\prime }} \end{array}$$ (19b)

## 5 Experiments and Results

We generate the NoC solution for two sample cases: the first one is a benchmark Triple Video Object Plane Decoder SoC [8] with 38 cores and 48 traffic flows. We generate an area-optimized topology against this benchmark without any QoS requirements to test our solution on a large application. The second is a multimedia SoC design with over 50 masters/slaves [5]; for the topology generation we target the backplane with 12 master and 4 slaves to a total of 21 flows, of which four flows are priority flows for which we convert latency guarantees from hop-counts to time units. For our topology generation purposes we set the data bit-width to 8 bits, and select the lowest possible frequency which satisfies the bandwidth requirements of the node with heaviest traffic flows for each SoC and generate solutions for Type A architectures.

For developing the MILP model, we used the Gusek software and from it generate an MPS file which was then solved using the Gurobi solver. We ran the software in a virtual machine inside a core-i5 system and the solver itself ran on two threads.

The results are displayed in Tables 1–6. As it can be seen in Tables 1 and 2, the overall solution cost is very low. Although we do not generate the number of slices in the result to keep the result platform independent, the resulting LUTS and Slice registers would be synthesized to a very area efficient solution for FPGA platforms in terms of area resources available. Tables 1 and 4 show that proposed mathematical model gives the solution in a very timely manner for both applications. Tables 2 and 5 show the outputs in terms of output links rather than routers as we directly model the links in our mathematical model. Tables 3 and 6 further show the per flow output link assignment generated for application-I and application-II through our respective proposed models.

**Table 1:** Result parameters for Application 1.

**Solution time (Sec)**	**10.16**
**Total Solution cost (LUT+Reg)**	2184
**Number of slice LUTs**	1360
**Number of slice registers**	824
**Bit width**	8
**Freq (Mhz)**	202.5

**Table 2:** Assigned output link details for Application 1.

**Output link no.**	**No of flows**	**Assigned Traffic to Link Capacity(%)**
1	4	88.0247
2	4	84.9383
3	4	97.9012
4	6	99.321
5	6	98.642
6	5	99.6296
7	11	99.8765
8	8	99.0741

**Table 3:** Per flow output link assignments for Application 1.

**Flow No**	**Traffic bandwidth of flow (mb/s)**	**Output link No utilized**	**Flow No**	**Traffic bandwidth of flow (mb/s)**	**Output link No utilized**
1	70	8	25	16	7
2	362	8	26	540	4
3	362	8	27	126	6
4	362	8	28	300	4
5	49	8	29	313	4
6	27	8	30	313	4
7	357	8	31	94	5
8	353	7	32	500	3
9	16	8	33	70	5
10	540	7	34	362	3
11	126	7	35	362	3
12	300	7	36	362	3
13	313	6	37	49	4
14	313	6	38	27	7
15	94	7	39	357	2
16	500	6	40	353	2
17	70	7	41	16	7
18	362	6	42	540	2
19	362	5	43	126	2
20	362	5	44	300	1
21	49	7	45	313	1
22	27	7	46	313	1
23	357	5	47	94	4
24	353	5	48	500	1

**Table 4:** Result parameters for Application 2.

**Solution time (Sec)**	2.7
**Total Solution cost (LUT+Reg)**	907
**Number of slice LUTs**	566
**Number of slice Registers**	341
**Bit width**	8
**Freq (Mhz)**	212.5

**Table 5:** Assigned output link details for Application 2.

**Output link No.**	**No of flows**	**Assigned Traffic to Link Capacity(%)**
1	5	84.4118
2	7	97.3529
3	8	97.3529
**P2P connection**	1	0.294118

**Table 6:** Per flow output link assignments for Application 2.

**Flow no**	**Traffic bandwidth of flow (mb/s)**	**QoS indicator**	**Output link No utilized**
1	5	1	3
2	5	1	2
3	600	0	3
4	60	0	3
5	720	0	3
6	50	0	3
7	690	0	2
8	330	0	2
9	60	0	3
10	240	0	2
11	540	0	1
12	120	0	3
13	180	0	2
14	40	0	3
15	300	0	1
16	120	0	2
17	500	0	1
18	5	1	1
19	90	0	2
20	5	1	N/A (P2P)
21	90	0	3

For both applications, the assigned link bandwidth compared to its total capacity is very high. We also note that for the second case, which is where we apply QoS requirements, one flow is not multiplexed with other flows in a router due to the QoS parameters preventing two priority flows in one router. As it is a single remaining flow, we replace the network on chip router with a point-to-point connection to further reduce cost. The results given in Table 5 supports the effectiveness of our proposed methodology with reference to QoS.

Another interesting result is that the synthesized frequency for both the applications is quite low. For the second application, it is around 30% lower than the result in [5]. Having low frequency of operation from the generated solutions is beneficial for the power costs of the application.

An important point from our experiments is that even for the application with 48 flows the results are computed in 10.16 seconds. This shows that for practical SoC applications we can use a MILP formulation to generate application specific network on chip topologies without resorting to heuristics. This is in counter to previous examples in literature which state the large times to generate results. For example, the time for Application 2 as shown in [5] varies from over one hour to over 32 hours. We contend that with the right MILP model formulation and a high quality commercial solver, MILP based solutions for application specific network on chips can be found for practical applications without waiting the result time going to hours.

## 6 Conclusion

In this paper, we highlighted the need for having a methodology to design area-optimized application specific network-on-chips (NoC) which also provides hard quality of service (QoS) guarantees. The proposed solution consisted of three main contributions (i) We proposed a novel NoC architecture which helps to provide the mechanism of latency guarantees and throughput regulation while adapting to variations in the application traffic (ii) The proposed system presented an optimal formulation for generating area-optimized NoCs while providing QoS guarantees for real time flows and extended it to support two NoC architectural models. The results demonstrated the effectiveness of proposed methodology on two experiments showing area-optimized NoC solutions for benchmark and industrial applications (iii) The results also showed that proposed MILP formulations have generated optimal solutions in a few seconds for a large application without resorting to heuristics or relaxations.
